# Development and validation of a patient-reported-experience measure to ascertain treatment satisfaction in migraine: the MISAT-Q questionnaire

**DOI:** 10.1186/s41687-025-00979-x

**Published:** 2025-12-15

**Authors:** Ana B. Gago-Veiga, Nuria González-García, Javier Díaz-de-Terán, Patricia Heredia-Rodríguez, Beatriz Armada-Peláez, Carlota Moya-Alarcón, Javier Soto-Álvarez, Javier Rejas-Gutiérrez, Miguel Á. Ruiz-Díaz

**Affiliations:** 1 Headache Unit, Hospital Universitario La Princesa, Madrid, Spain; 2 Group for the Study of Headache, Spanish Society of Neurology (GECSEN), Madrid, Spain; 3 Headache Unit, Hospital Clínico San Carlos, Madrid, Spain; 4 Headache Unit, Hospital Universitario La Paz, Madrid, Spain; 5 Group for the Study of Headache, Spanish Society of Nursing Neurology (GECSEDENE), Valencia, Spain; 6 Medical Department, Pfizer S.L.U., Alcobendas, Madrid, Spain; 7 Health Outcomes Research Department, Pfizer S.L.U., Alcobendas, Madrid, Spain; 8 MEVAFARMA, Universidad Carlos III, Madrid, Spain; 9 EACCOS Research Group, School of Psychology, Universidad Autónoma de Madrid, Madrid, Spain; 10 Department of Social Psychology and Methodology, School of Psychology, Universidad Autónoma de Madrid, Iván Pavlov, 6, Fuencarral-El Pardo, Madrid, 28049 Spain

**Keywords:** Migraine, Treatment satisfaction, MISAT-Q, Daily medical care, Validation, Development, Psychometric properties, Self-report instrument, Patient-reported experience measure (PREM)

## Abstract

**Background:**

The patient’s perspective is increasingly relevant in health decision making. In daily practice, care of patients with migraine should include evaluation of their satisfaction with acute and preventive treatments, the interference with daily living, as well as with the care received by the healthcare team. The aim was to develop and validate a novel specific self-reported patient-reported-experience measure (PREM) in Spanish (Spain), to ascertain satisfaction with migraine treatment; the MISAT-Q questionnaire.

**Methodology:**

A multicenter, crosssectional, non-interventional study was conducted between June 2023 and March 2024 in headache units in Spain. Patients with migraine currently receiving treatment were included. A 35-items preliminary instrument was developed after literature review and cognitive debriefing with experts and patients. Three samples were used: a) 23 patients to assess feasibility/pertinence of items; b) 158 patients for item reduction; and c) 209 patients for validation of PREM. Measures for concept validity included Patient Global Impression of change (PGI), Headache Impact Scale-6 items (HIT-6), and Health-Related Quality-of-life (EQ-5D-3 L). Feasibility, reliability and validity (content, discriminant, construct, and concurrent) were assessed.

**Results:**

Feasibility: face value (good acceptance to answer the questionnaire), administration time (below 10 minutes), floor and ceiling effects (below 40% in any extreme response category), percentage of missing values in each item (below 20%) was found in the validation sample. Factor analysis item reduction resulted in a 22-item questionnaire with 7 dimensions: undesirable side effects, treatment effectiveness in crisis and in prevention, convenience-of-use, impact on daily activities, medical care, and global satisfaction, supported with confirmatory factor analysis: CFI = 0.983; TLI = 0.980; χ^2^/df = 1.808; RMSEA = 0.075. Reliability was high: Cronbach’s alpha = 0.90 and intraclass-correlation coefficient = 0.95. Dimensions of questionnaire showed significant and moderate convergent correlations with the overall score (0.58–0.68, *p* < 0.001). Concurrent validity with HIT-6 showed correlation’s coefficients ranging from −0.19 to −0.48 (*p* < 0.01 in all cases, except medical care) and with EQ-5D-3 L (correlations from 0.17 to 0.39, *p* < 0.05 in all cases, except medical care). Differences in satisfaction were found according with migraine severity (F = 6.73, *p* = 0.002), as well as in HIT-6 scores (F = 8.20, *p* < 0.002). MISAT-Q discriminated overall satisfaction between patients with worse, no change and better change with treatment (F = 15.85; *p* < 0.001).

**Conclusions:**

The MISAT-Q questionnaire is a well-accepted, reliable and valid measure of migraine treatment satisfaction in Spanish. Responsiveness attribute needs to be explored. This novel PREM may facilitate clinicians when making health decision in the treatment management of patients with migraine.

**Supplementary information:**

The online version contains supplementary material available at 10.1186/s41687-025-00979-x.

## Background

Migraine is a highly prevalent chronic neurological disease, being the second leading cause of disability and affecting numerous individuals, families, work, and society [1–4]. Individuals afflicted with migraine may consult with a variety of healthcare providers. However, those with higher disability grades are more likely to seek consultations with specialists, while those with lower grades typically visit primary care or general practitioners, or alternatively, pharmacists [5, 6]. In all cases, the pharmacological management of migraines should be individualized and tailored to the needs of each patient. This is due to the fact that dual management of severe migraine treatment involves a combined strategy to prevent attacks and treat acute episodes when they occur [7]. In recent years, significant advancements have been made in the therapeutic management of migraines, encompassing both symptomatic and preventive approaches. These developments have led to substantial improvements in patient health-related quality of life (HRQoL) [7, 8]. However, a significant proportion of patients do not receive adequate treatment or are not satisfied with their treatment, as evidenced by the OVERCOME study [5, 6, 9]. The management of migraine is a complex matter that has been a subject of concern. It is imperative to enhance the management of these difficult-to-treat patients, as inadequate migraine control can escalate the likelihood of medication overuse or chronic migraine [8].

The patient’s perspective is of increasing relevance in medical decision-making, particularly among individuals suffering migraines [10]. In daily practice, the management of patients with migraines should encompass a systematic evaluation of their satisfaction with acute and preventive pharmacological treatment, as well as with the care received from the healthcare team and its impact on their daily lives [11]. Consequently, it becomes imperative to consider patient perspectives and promote the use of patient-reported outcomes (PROs). This concept is particularly notable in the context of headache disorders and migraines [11]. These instruments have been used in research and clinical practice to enhance patient management and facilitate shared decision-making, incorporating the patient’s perspective [12]. There are important tools for capturing information from the patient’s perspective and they should be used to evaluate the impact on the HRQoL, functional and emotional disability and, specifically in migraine, to evaluate the course of patients after initiating preventive treatment. Some Patient-Reported-Outcomes Measures (PROMs) are already available in clinical practice: HIT-6 (Headache Impact Test), the MIDAS scale (Migraine Disability Assessment Scale), the MSQ (Migraine-Specific Quality of Life Questionnaire), the MTAQ (Migraine Therapy Assessment Questionnaire), or Patient-Reported-Experience Measures (PREMs) assessing patient satisfaction with migraine treatments such as the Patient Perception of Migraine Questionnaire (PPM-Q), its revised version (PPMQ-R) or the Migraine Treatment Satisfaction Measure (MTSM) [13–19]. Despite their reliability and accuracy, instruments such as these are not without their limitations. They are effective tools for measuring patient satisfaction with various migraine treatment options and can provide valuable information on the impact of migraines on patients’ daily lives. However, it is important to note that such instruments do not capture the entire spectrum of patient satisfaction with therapy. The Migraine Treatment Satisfaction Measure (MTSM) is not a measure of treatment effectiveness or efficacy. The Patient Perception of Migraine Treatment Questionnaire-Revised (PPMQ-R) evaluates the level of patient satisfaction with treatment for acute migraine attacks in five areas. The following factors were considered in the analysis: adverse effect discomfort, ease of use, cost, functionality, and efficacy. It should be noted that the analysis did not include prevention, medical care, or overall satisfaction. In addition, despite the valuable information regarding treatment effectiveness that these tools provide, they may not encompass all aspects of a patient’s satisfaction with their treatment. It is therefore recommended that several questionnaires be utilized in conjunction if we wish to cover all the pertinent dimensions and to obtain a more comprehensive evaluation of the treatment [11, 12].

Consequently, there is still a need for instruments that assess satisfaction with migraine treatments in a comprehensive manner, taking into account the unique characteristics of migraine treatment. This assessment should particularly differentiate recent migraine treatments and the medical care received, as these factors have been demonstrated to significantly influence patient satisfaction. The objective of this study was to develop and validate a novel multidimensional PREM to assess satisfaction with migraine treatment in Spanish (Spain). The MISAT-Q questionnaire was developed to fulfill this objective.

## Methods

### Ethics

This study was conducted in accordance with the principles of Good Clinical Practice and Good Laboratory Practice as referenced in the International Council for Harmonization guidelines, as well as all applicable regulations, including any institutional review board requirements relevant to clinical studies. The study also complied with the recommendations in the most recent version of the Declaration of Helsinki. Participants provided written informed consent before any study-related procedures were performed. The protocol was approved by the institutional review board of Hospital Universitario de La Princesa, Madrid (Spain), and the Clinical Research Ethics Committee of Universidad Autónoma de Madrid, Spain (register code CEI-131–2722). The manuscript was prepared according to the COSMIN reporting standards for developing, evaluating, and refining patient-reported or clinical measurement instruments (see Supplementary Information with COSMIN checklist dully completed) [20].

### Study design, settings and participants

A multicenter, crosssectional, non-interventional study was conducted between June 2023 and March 2024 at three Headache Units in Madrid (Spain): Hospital Universitario de La Princesa, Hospital Clínico San Carlos and Hospital Universitario La Paz. Patients were sequentially selected among those who met the following selection criteria: outpatients ≥18 years of age, diagnosed with episodic ( < 15 monthly headache days [MHDs]) or chronic migraines (≥15 MHDs for more than three consecutive months) according to the International Classification of Headache Disorders (ICHD-3) [6], initiating or following acute or preventive migraine treatment at the time of screening, able to understand and answer the health questionnaires included in the study (Spanish versions, see Supplementary Information with a brief description of the questionnaires used), and willing to sign the informed consent form. Patients participating in any clinical trial regarding migraine treatment were excluded.

Four different samples were used: 1) *pilot-content selection sample*: comprised of 23 randomly recruited patients; 2) *reduction sample*: 158 patients under migraine treatment; 3) *validation sample*: 209 patients selected using the same criteria as in the reduction sample; and 4) *retest sample*: 32 patients were selected from those in the validation sample, and answered the questionnaire one week apart (approximately) from the first administration, which was considered a time interval sufficient to test temporal stability of instrument (shorter for any change in health status, but longer to avoid patients remembering the narrative of items asked in the first administration). Patient selection was random and sequential, until the indicated subject quotas by site were covered. Since there is no definitive consensus yet on the appropriate size of sample to develop a new PREM, the study followed various existing guidelines or recommendations in the literature to calculate the samples sizes for the different phases of PREM development (the Supplementary Information provides additional information on this matter) [21–23].

### Questionnaire

The questionnaire encompassed three main phases: content validation and item generation (preliminary or first version), item reduction of the first version to produce the final version and psychometric validation phase of the final version.

#### Content validation and item generation

The questionnaire process began with the selection of a panel of experts composed of three neurologists, a nurse specialized in headaches, an expert patient, three health-outcomes-research specialists, and a methodologist, who supervised all phases of the questionnaire process and validation. Content extraction included a literature review of articles published on satisfaction and treatment satisfaction. A review and compilation of existing questionnaires on satisfaction with treatment were carried out and the SATMED-Q model was used as a reference theoretical structure framework [24]. The panel of experts generated an initial series of questions in Spanish (Spain) relating to the following aspects: undesirable side effects, acute effectiveness, prevention effectiveness, overall satisfaction, convenience of use, expectations, available clinical options, recommendation disposition, adherence, satisfaction with medical care, impact on daily life, emotions, and beliefs about treatments. Content validation implied thirteen migraine content-specialists (8 neurologists, 2 nurses, 1 psychologist and 2 health-outcomes-research specialists) scored each of the proposed items in all the dimensions measured and defined to scaffold the questionnaire (1 = measured, 0 = unsure, −1 = not measured). Item-Domain congruence was measured using the Hambleton-Rovinelli procedure for assessing item validity by inter-rater agreement [25]. Items were worded in affirmative phrasing and were designed to ensure that they referred to a single concept, intending to be easy to answer. Possible answers were scored using a 5-point Likerttype scale: 0 = “No, not at all”; 1 = “A little bit”; 2 = “Neither a lot, nor a little”; 3 = “Quite a lot”; 4 = “Yes, very much”. Complete agreement between content specialists was required, resulting in a preliminary version of the questionnaire with 35 items and eight dimensions. This version was self-patient administered to the pilot-content selection sample, for assessment pertinence of possible items, missing contents, concept convenience, wording adequacy, and relevant concepts which might be missing or the presence of any difficulty found in answering the items or the presentation format.

#### Item reduction

The initial 35-item questionnaire was administered to the reduction sample, using an anonymized electronic format. The information obtained was used: (1) to check adjustment of the patient responses to the proposed structure (dimensions or subscales); (2) to assess the metric properties of the items; and (3) to reduce the number of questions. The questionnaire was reduced and underlying dimensions were determined via a sequence of exploratory factor analyses (EFAs), based on the assessment of internal consistency [26]. Internal consistency was evaluated by means of Cronbach’s alpha reliability coefficient [23]. More information regarding the reduction of the length of the questionnaire and analysis of dimensionality may be found in the Supplementary Information.

#### Psychometric properties testing of the final version

The reduced version (final version, see the Supplementary Information including the MISAT-Q final version) was included in the validation phase of the study to test its psychometric properties, together with clinical information of relevance for the patient, sociodemographic information, and the following PROMs: Headache Impact Scale-6 items (HIT-6), Health-related quality-of-life (HRQoL) EuroQoL (EQ-5D-3 L), and Patient Global Impression scale (PGI), all of them in their Spanish validated versions [12, 27, 28]. The Supplementary Information includes a brief description of these scales. The data obtained from this sample were then used for the following: (1) to ratify the structure of the abridged questionnaire through confirmatory factor analysis (CFA) [29], (2) to assess the metric properties of the questionnaire [23], and (3) to create correction norms for the Spanish population. The following metric properties were studied: (1) *feasibility:* administration time, floor and ceiling effects, percentage of missing values in each item; (2) *reliability*: internal consistency, evaluated by means of Cronbach’s alpha coefficient and the Pearson correlation coefficient between items and between each item and the total composite score; testretest (temporal stability), evaluated by correlating two administrations of the questionnaire based on the Pearson correlation coefficient and intraclass correlation coefficient; and Standard Error of Measurement (SEM), calculated as the product of standard deviation multiplied by the square root of 1 minus the value of Cronbach’s alpha coefficient [23, 30]; (4) *construct validity*: the structure in dimensions of the answers gathered with the final questionnaire was established by CFA [29]; (5) *concurrent validity*: correlations between the HIT-6 scale and the EuroQoL VAS scores were assessed; (6) *divergent validity*: correlations with EQ-5D-3 L scores were expected to be lower than between questionnaire dimensions; and (7) *discriminant validity*: the capability of each subscale to discriminate between the 25% of subjects with the lowest scores and the 25% with the highest scores (established from the total composite scale scores) was analyzed. Comparisons were also made between groups known to behave differently based on PGI scores grouped in 3 levels: very much worse, much worse = worse; minimally worse, no change, minimally improved = no change; much improved, very much improved = better.

### Statistical analysis

This is the primary analysis of these data. Statistical testing was two-tailed and considered statistically significant at p-value < 0.05, when appropriate. Statistical analysis included descriptive statistics of both reduction and validation samples. EFA was applied to analyze the dimensional structure of the questionnaire and to estimate item commonalities in the reduced version of questionnaire [21, 22, 26]. Two extraction methods were used: principal components and principal axes, and two rotation methods (varimax and oblimin). Heuristics for determining the optimum number of factors comprised the Kaiser K1 rule, scree test, the percentage of variance accounted for, and the magnitude of the eigenvalues after rotation. Moreover, a number of decision rules were applied according to published literature on this matter [31–36] (See Supplementary materials for a more detailed explanation). CFA was carried out including all the refined subscales of the final version to check that the structure remained stable, and to assess that adequate goodness-of-fit was attained. Standard criteria for the goodness-of-fit statistics were used: Comparative fit index (CFI > 0.95), Tucker-Lewis index (TLI > 0.95), chi-square to degrees of freedom ratio (χ^2^/df < 2), and root mean square error of approximation (RMSEA < 0.08) [29]. Linear correlation coefficients were estimated between the final version of MISAT-Q and the other PROMs used to analyze its concurrent validity [23]. ANOVA and independent *t*-test were used to compare satisfaction scores, overall or in dimensions, between groups for discriminant purposes or known groups testing.

Latent Class Analysis (LCA) was performed to validate the suitability of dimension scores to describe groups of patients existing in the sample gathered. The maximum likelihood method was used and dimension scores were defined as cluster indicators, while HIT-6 scores (inverted), type of migraine (categorical) and absence of adverse effects (dichotomous) were included as covariates. The number of latent classes was incremented until attaining a non-significant goodness-of-fit (GOF) statistic. All variable scores were standardized to a 0–1 scale to plot profiles. Suspecting that any of the dimensions could not be capable of discriminating between groups of patients, 4 groups of patients were defined based on the total score split by quartiles, and a repeated measures ANOVA was performed to check whether significant differences were detected within each dimension.

The total composite scores were transformed to a more intuitive and easier to understand metric, with a minimum of 0 (no satisfaction) and a maximum of 100 (best possible satisfaction), using the following expression: Total transformed composite score = [Total score obtained by the patient – Minimum possible score]/[Maximum possible score – Minimum possible score] × 100, where the maximum total score = 88 and the minimum total score = 0). A similar expression can be used to change the metric of each individual dimension/subscale. The side-effects satisfaction dimension metric needed to be inverted when scoring to apply this transformation, both when calculating the total composite score and the dimension alone as well. To promote enhanced interpretability, correction norms were developed. These correction norms enabled the transfer of the observed metric 0–100 scores to the corresponding deciles in the normative sample.

All statistical analyses were performed using the IBM SPSS version 29.0 statistical package (IBM Corp., Armonk, NY, USA), AMOS 29.00 (IBM Corp., Armonk, NY, USA), Mplus 8.1 (Muthén & Muthén, Los Angeles, CA, USA) and LantentGold v8 (Statistical Innovations Europe, Tilburg, The Netherlands).

## Results

Table 1 reports sociodemographic and clinical information for the reduction and validation samples (Supplementary Information, Fig. S1 and Table S1 include age distribution and number of migraine episodes in the previous four weeks). The amount of missing data was minimal and completely random, with data lacking in 10% or fewer of patients enrolled in the study (see Supplementary Information, Table S2). Consequently, the process of value imputation for missing data was not implemented. Descriptive statistics regarding the dimension scores and the total composite score can be found in Supplementary Information (Table S3).

**Table 1:** Demographic and clinical characteristics of patients included in the study

	Reduction sample (*n* = 158)	Validation sample (*n* = 209)
Age: mean (SD)	43.9 (13.0)	46.6 (12.9)
Sex, male: n (%)	134 (83.8)	172 (89.6)
Migraine Type: n (%) Low frequency-episodic ( < 10 MHD) High-frequency episodic (10–14 MHD) Chronic (≥15 MHD)	25 (15.6) 40 (25.6) 93 (58.8)	48 (23.0) 37 (17.7) 107 (51.2)
Unknown	-	17 (8.1)
Number of MHD: mean (SD)	11.4 (7.2)	11.5 (8.8)
Aura, yes: n (%)	59 (36.9)	66 (34.4)
Years from last treatment: mean (SD)	1.7 (3.1)	1.7 (3.1)
HIT-6: mean (SD)		61.4 (7.6)
Small impact: n (%)		13 (6.7)
Moderate impact: n (%)		23 (11.9)
Severe impact: n (%)		33 (17.1)
Very severe impact: n (%)		124 (64.2)
Missing data: n (%)		16 (7.7)
EQ-5D-3 L: utility mean (SD)		0.686 (0.223)
HRQoL VAS: mean (SD)		63.9 (21.2)
Missing data: n (%)		3 (1.4)
PGI: n (%)		
Very much better		13 (6.4)
Much better		35 (17.2)
Somewhat better		51 (25.0)
No change		61(29.9)
Somewhat worse		30 (14.7)
Much worse		14 (6.9)
Very much worse		0 (0%)
Missing data: n (%)		5 (2.4)

MHD: Migraine Headache Days. SD: Standard Deviation

### Content validation and piloting

Content specialists scored the pertinence and adequacy of each item for measuring the pertinent domain, as compared to each one of the competing domains (concepts) proposed for the structure of the questionnaire. All 35 items obtained a positive item-domain score in the theoretical intended dimension, ranging between 0.52 and 0.92 (88.0% of values ≥0.70). The following pattern was observed: the overall satisfaction dimension tended to obtain low positive scores for most items. Similarly, impact on the QoL domain tended to obtain moderate positive scores on most items, particularly for side-effect, prevention and emotion items. Content-specialists agreed on the assignment of items to their target construct dimension as the main measurement dimension. However, two dimensions presented some minor positive values in other competing theoretical dimensions: overall satisfaction, prevention and emotions. This result was expected since the treatment satisfaction construct assumes dimensions to be conceptually correlated.

### Item reduction

Patient answers (148 respondents) showed a very small number of missing responses, with 89.2% to 99.3% of complete answers by dimension (see Table S2 in Supplementary Information). The necessity of imputation was not pursued. Dimension scores were computed as the average score of existing item responses in the dimension.

Results suggested that the original set of 35 items could be reduced to a final version with 22 items without decreasing reliability and suggesting the possible unfolding of the convenience of use dimension. Table 2 reports the internal consistency results before and after item reduction. Values obtained for Cronbach’s alpha coefficient suggested good-to-excellent internal consistency (α≥0.80), except for convenience of use (α = 0.56). The percentage of variances accounted for by the first dimension of each subscale suggest that the subscales are unidimensional. One of the proposed dimensions, emotions and feelings, was discarded by the expert panel. Item commonalities were very low for two items ( < 0.3), dimension-explained variance was low (38%) and Cronbach’s alpha was low (α = 0.60). Discriminant validity was also questionable, since factor loadings were smaller than some correlations with other dimensions.

**Table 2:** Reduction sample: internal consistency of subscales

Dimensions	Number of items		Cronbach’s alpha		% variance explained^*^
	Initial	Final	Initial	Final	
Undesirable side effects	4	3	0.91	0.93	79.1
Efectiveness in crisis	3	3	0.83	0.83	74.8
Efectiveness in prevention	6	4	0.94	0.94	77.4
Convenience of use	5	3	0.30	0.56	34.3
Impact on daily activities	4	3	0.94	0.96	85.4
Medical care	6	3	0.795	0.80	55.5
Emotions	3	0	0.60	-	56.5
General satisfaction	4	3	0.52	0.80	54.2
Total Score	35	22	0.90	0.92	-

*Percentage of variance accounted for by the first factor in each subscale

While factor analysis of items isolated by dimension showed to be unidimensional after item reduction, the joint factor solution for all items considered together raised high correlations between dimension factor scores (Table 3), suggesting cross-loadings between dimensions. The general satisfaction dimension correlated significantly with all other dimensions (*p* < 0.05), especially with effectiveness in crisis (*r* = 0.59), effectiveness in prevention (*r* = 0.68) and impact on daily activities (0.56). The dimension correlating less with other dimensions was medical care, showing the higher correlation with convenience of use (*r* = 0.39), not correlating with undesired side effects (*r* = 0.08), effectiveness in crisis (*r* = 0.13) nor with effectiveness in prevention (*r* = 0.01). Dimensions showed enough discriminant validity, and all factor loadings in the nominal dimension were higher than the correlation of the selected dimension with the remaining ones.

**Table 3:** Reduction sample: factor correlations

Dimension (F)	F1	F2	F3	F4	F5	F6	F7	F8
1. Undesirable side effects	1							
2. Efectiveness in crisis	−0.366^**^	1						
3. Efectiveness in prevention	−0.406^**^	0.700^**^	1					
4. Convenience of use	−0.213^**^	0.266^**^	0.105^†^	1				
5. Impact on daily activities	−0.451^**^	0.651^**^	0.761^**^	0.156	1			
6. Medical care	−0.082^†^	0.130^†^	0.014^†^	0.394^**^	0.173^*^	1		
7. Emotions	−0.559^**^	0.589^**^	0.601^**^	0.330^**^	0.614^**^	0.210^*^	1	
8. General satisfaction	−0.315^**^	0.593^**^	0.683^**^	0.203^*^	0.561^**^	0.218^**^	0.516^**^	1

** *p* < 0.01, * *p* < 0.05, ^†^*p* > 0.05

## Validation study

### Feasibility

The nonresponse rate in the validation sample (209 patients) was very low: 79.0% of patients answered all the questions in the questionnaire and those omitting answers did so by not answering all items in one or two dimensions. Subscales with lower response rates were medical care (88.0%) and convenience of use (90.0%). Average response time for the 22-item version was 5.7 minutes (SD = 3.3) as computed from the test-retest sample.

The total composite scores exhibited a negative skewed distribution, with a mean (SD) of 74.9 (16.9). The median was 77.1. The minimum recorded score was 29.8 (0.5% of cases), and the maximum 100 (1.0%). The responses, for all items, were distributed along all the proposed response categories. Except for items in the *undesirable side effects* subscale, which is inverted in meaning, the distribution of the responses showed a slight negative skewness; the item with the most skewed distribution (“*My medical team has informed me about my migraine and its treatment”*) accumulated 78.0% of the responses in the upper part of the scale. All distributions were unimodal. The subscale *undesirable side effects* accumulated the responses in the lower portion of the scale: between 53.0% and 61.0% of responses were in the category “No, not at all”. This floor effect was also found in the reduction sample and, in our opinion, justifies the possibility of using this subscale independently from the rest as an indicator of situations of lack of tolerability.

### Reliability

Cronbach’s alpha coefficient in the validation sample was high; α = 0.78 or above for all subscales (Table 4), except for the dimension convenience of use (α = 0.43). The total composite scale attained a value of α = 0.90. Excepting the convenience dimension, the first eigenvalue was markedly bigger than the second on all subscales, and the first dimension of each subscale accounted for a percentage of variance between 70.0% and 90.0%, thus indicating that the subscales behave in a unidimensional manner. Test-retest correlation for the total score was very high (*r* = 0.88) when administering the questionnaire a second time—5.8 (1.6) days later on average. Table 4 shows test-retest reliability statistics with high two-facet intraclass-correlation coefficients (ICC) for the total composite score, 0.95. The mean total composite score was 86.8 (SD = 12.8) for the first administration, and 91.5 (SD = 14.1) for the second. The difference between these means was not significant (*t* = 0.69, *p* = 0.502). SEM was 5.88 for the total composite MISAT-Q score.

**Table 4:** Validation sample: reliability statistics

	#items	Alpha	First eigenvalue	Second eigenvalue	% variance explained^*^	Test-Retest	
						r	ICC
Undesirable side effects	3	0.91	2.54	0.28	84.5%	0.61	0.897
Crisis effectiveness	3	0.78	2.09	0.50	70.1%	0.54	0.90
Prevention effectiveness	4	0.92	3.22	0.32	80.5%	0.93	0.93
Convenience of use	3	0.43	1.51	0.91	51.3%	0.75	0.71
Impact on daily activities	3	0.94	2.69	0.17	89.4%	0.92	0.97
Medical care	3	0.83	2.35	0.34	77.5%	0.66	0.76
Global satisfaction	3	0.82	2.21	0.44	72.9%	0.68	0.81
Total composite score	22	0.90	–	–	–	0.88	0.95

*Percentage of variance explained by the first factor in each subscale. r: Pearcon Correlation Coefficient. ICC: Intra-class correlation coefficient

### Construct validity

**Fig. 1:**
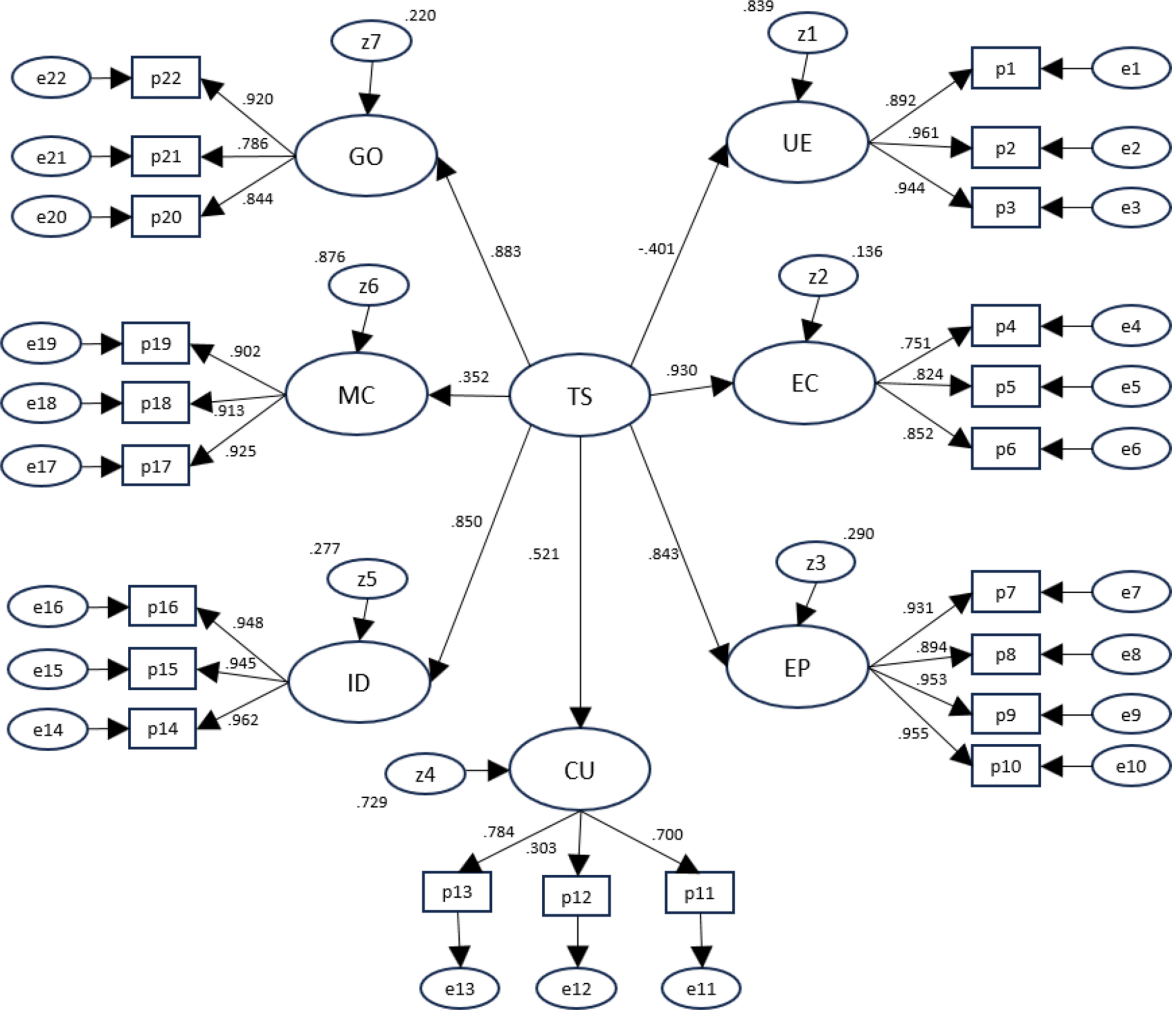
Confirmatory factor analysis standardized estimates. TS = Treatment Satisfaction, UE = Undesirable Side-Effects, EC = Effectiveness in Crisis, EC = Effectiveness in Prevention, CU = Convenience of Use, ID = Impact on Daily activities, MD = Medical Care, GO = General Opinions

Assessment of the dimensionality of the questionnaire was replicated using the reduced version and data obtained from the validation sample (22 items). Figure 1 shows the confirmatory factor analysis solution. All first order factor loadings were between 0.70 and 0.96, statistically significant (*p* < 0.001) and within admissible values, except for the loading of item 12 (“*It is very unlikely that I forget to take my medication*”) on the undesired effects dimension (λ = 0.30; *p* = 0.017). No cross-loadings were allowed. Second order loadings ranged between 0.35 and 0.93 (in absolute value) and were all significant (*p* < 0.001). Goodness-of-fit statistics indicated a good or very good fit: CFI = 0.983; TLI = 0.980; χ^2^/df = 1.808; RMSEA = 0.075 (95% CI = 0.059–0.082).

### Concurrent validity

Questionnaire scores significantly correlated with the scores on the HIT-6 (Table 5): a correlation of −0.51 was obtained between the total composite score and impact on daily activities domain, with correlations ranging from −0.18 to −0.51 (*p* < 0.01 in all cases), except medical care (*r* = 0.02, *p* = 0.482). HIT-6 impact levels attained significant differences in average overall satisfaction scores (F = 13.84, *p* < 0.001), while the very severe- and small-impact groups were significantly different from all other groups (*p* < 0.05), the moderate- and severe-impact groups did not differ from each other (*p* = 0.986).

Correlation with the scores of the EQ-5D (Table 5) were also significant: a correlation of 0.41 was obtained between the total composite score and utility scores, with correlations ranging from 0.27 to 0.43 (*p* < 0.05 in all cases), except medical follow-up (*r* = 0.04, *p* = 0.275). Furthermore, MISAT-Q, total score and dimension scores significantly correlated with the VAS of EQ-5D scores (Table 5). The correlations proved significant for both the total composite score (*r* = 0.33) and for all the subscales, except for *medical care* (*r* = 0.06, *p* = 0.232).

**Table 5:** Correlations between MISAT-Q dimensions and concurrent measures

Domain	With	Statistic			
		Correlation	Count	Lower C.I.	Upper C.I.
Side Effects	HIT6	−0.36	168	−0.49	−0.23
	EQ5D_U	0.29	164	0.14	0.42
	VAS	0.14	169	−0.01	0.29
Effectiveness Crisis	HIT6	−0.42	168	−0.54	−0.29
	EQ5D_U	0.27	164	0.12	0.41
	VAS	0.25	169	0.10	0.39
Effectiveness Prevention	HIT6	−0.42	168	−0.53	−0.28
	EQ5D_U	0.31	164	0.16	0.44
	VAS	0.24	169	0.09	0.37
Convenience	HIT6	−0.18	164	−0.32	−0.02
	EQ5D_U	0.22	160	0.07	0.36
	VAS	0.18	165	0.03	0.33
Impact Daily Activities	HIT6	−0.51	166	−0.62	−0.39
	EQ5D_U	0.43	162	0.30	0.55
	VAS	0.43	167	0.29	0.54
Medical care	HIT6	0.02	166	−0.14	0.17
	EQ5D_U	0.04	163	−0.11	0.19
	VAS	0.06	167	−0.09	0.21
General Satisfaction	HIT6	−0.31	167	−0.44	−0.17
	EQ5D_U	0.27	163	0.13	0.41
	VAS	0.21	168	0.06	0.35
Total Score	HIT6	−0.50	168	−0.61	−0.38
	EQ5D_U	0.41	164	0.27	0.53
	VAS	0.33	169	0.19	0.46

C.I. : Confidence Interval, level: 95%, EQ5D_U: Utility index of EQ-5D questionnaire, VAS: Visual analogue scale of EQ-5D questionnaire

### Discriminant validity

Comparison of the overall satisfaction scores between the three PGI levels yielded significant differences between health improvement groups (F = 15.85; *p* < 0.001) with the following averages (Table S4, Supplementary Information): worse = 59.8 (SD = 14.0), no change = 73.5 (SD = 17.4), improved = 84.5 (SD = 16.9), with all averages being significantly different (*p* < 0.006). Average differences in MISAT-Q scores between severity groups were greater than 1.96 times the value of SEM.

In order to analyze the discriminative capacity of each item considered individually, two patient groups were created from the scores obtained in the total composite scale. The first group was comprised of the 25.0% of patients with the lowest scores (quartile 1), while the second was comprised of the 25.0% of patients with the highest scores (quartile 4). Comparison between these two groups yielded significant differences for all items (*t*(104) > 2.99 and *p* < 0.002) except for the item related to forgetting medication intake (*t* = 2.25, *p* = 0.01).

### Known groups validity

No significant differences were found between women and men in the overall satisfaction score (*t* = 0.16, *p* = 0.876) nor in the isolated subscales (*p* > 0.141 in all cases). Similarly, no significant differences were found between sex in HIT-6 scores (*t* = 0.50, *p* = 0.615), EQ-5D utilities (*t* = 0.02, *p* = 0.983) or VAS scores (*t* = 0.24, *p* = 0.407).

Significant differences in overall satisfaction were found between groups of patients with different diagnosed severity (F = 6.73, *p* = 0.002), as well as in HIT-6 scores (F = 8.20, *p* < 0.002), EQ-5D utilities (F = 14.22, *p* < 0.001) and VAS scores (F = 16.73, *p* < 0.001). The average satisfaction score in the ≥15 MHD group (M_chronic_ = 71.1) was significantly different from the < 10 MHD group (M_low-freq_ = 82.2, *p* = 0.001) but the 10–14 MHD group of patients (M_high-freq_ = 77.1) did not differ from the two extreme groups (*p* = 0.183 and *p* = 0.386, respectively). No differences were found in overall satisfaction between those patients with and without aura (*t* = 0.88, *p* = 0.379).

## Interpretability

The correction norms are shown in Table 6. These norms allow us to transfer the observed metric 0–100 scores to the corresponding deciles in the normative sample. To illustrate, a patient with a total composite satisfaction score of 73 would be situated in decile 4, indicating that 40.0% of patients in the population report similar or lower overall satisfaction levels.

**Table 6:** MISAT-Q total composite deciles

Deciles	Total	
	Min.	Max.
1	29.8	45.8
2	46.4	62.5
3	63.1	69.1
4	69.5	73.2
5	73.8	77.1
6	77.5	81.9
7	82.1	86.0
8	86.1	89.3
9	89.6	92.9
10	93.1	100.0

**Fig. 2:**
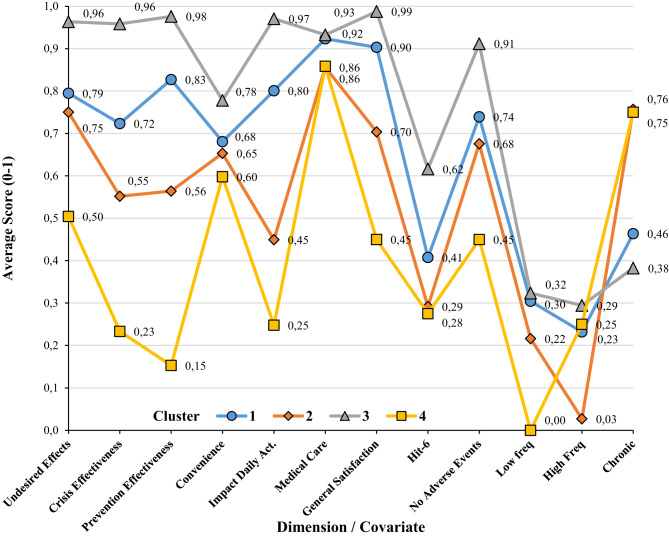
Latent class profiles by cluster. Average dimesnion scores in 0–1 scale, average inverted HIT-6 average scores, proportion of cases with absence of adverse events and propotion of cases in severity group (low frequency, high frequency and chronic)

Four clusters were identified as having different profiles in the LCA, while obtaining a non-significant GOF value (L^2^ = 3910.69, *p* = 0.10). Average dimension scores for the four latent profiles (clusters) appeared to describe four profiles with decreasing average values in the seven dimensions (Fig. 2), although not all dimensions were able to discriminate between profiles (see Supplementary Information, Tables S5 to S12). For example, cluster 3 contained 21% of patients, gathered those having higher scores in all dimensions and presented with fewer symptoms; 90% did not present adverse effects, and only 50% suffered chronic migraine. In the opposite extreme, cluster 4 contained 12% of patients, gathered those having lower scores in all dimensions, presented with more severe symptoms; 50% did not present adverse effects, and 80% suffered chronic migraine. All dimension scores were capable of discriminating between some of the clusters (*p* < 0.001), except comorbidity scores (*p* = 0.066) and follow-up (*p* = 0.11).

The seven dimensions demonstrated the capacity to differentiate between the quartile groups delineated by the total MISAT-Q score (*p* < 0.001, see Supplementary Information, Table S13), exhibiting a linear pattern for averages in all dimensions (*p* < 0.001). Specifically, medical care scores were able to distinguish between first quartile and forth quartile groups (*p* < 0.001, Supplementary Information, Tables S13–S15), and between Q1 and Q2 groups.

## Discussion

The objective of this study was to develop and assess the metric properties of a novel instrument in Spanish (Spain) to measure treatment satisfaction in patients with migraine, to be used in clinical practice with any specific migraine medication. The findings of this study demonstrated the validity, reliability, and feasibility of the MISAT-Q questionnaire, which is suitable for use as a standalone instrument, with its total composite score providing a comprehensive evaluation, and it can also be utilized to assess patient satisfaction with various aspects of treatment. The subscales of the MISAT-Q questionnaire have been demonstrated to be valid and reliable. The findings indicate that the MISAT-Q demonstrates optimal metric properties, aligning with the established COSMIN guidelines [20]. From a feasibility standpoint, the response rate is deemed to be highly satisfactory, and the administration time is reported to be very brief, with an average of five and a half minutes and a self-administered process. This renders the test highly feasible for use at any level of healthcare, particularly in primary care, where the time available for seeing patients is usually limited.

It has become increasingly recognized that the viewpoint of the patient should be taken into account when evaluating a medical treatment [37]. A significant domain of patient-centered evaluation entails the assessment of patient satisfaction with treatment, particularly in the context of migraine. This evaluation involves the analysis of patient progress following the initiation of treatment for migraine attacks and also as a preventative measure [12, 38, 39]. Treatment satisfaction recently appears to be increasingly used as a PREM when assessing new or existing treatments [39], despite the American Headache Society position statement on integrating new migraine treatments into clinical practice [40]. On the one hand, this is because satisfaction helps to appraise the goodness and convenience of the medication provided. Conversely, elevated levels of treatment satisfaction have been demonstrated to be associated with increased patient adherence to therapy and a greater patient desire to continue using the drug. This association may serve as a valuable predictor of treatment compliance and could potentially enhance the effectiveness of the administered therapy. Moreover, it has been suggested that this increased satisfaction may contribute to a reduction in the risk of migraine chronification or medication overuse [41]. This is particularly relevant since we know that adherence to classic preventive and acute treatments is very low among migraine patients [5, 6, 9, 41]. In fact, in recent years, regular assessment of treatment satisfaction is becoming more and more frequent in different pathologies involving different drugs (often using specific measurement instruments), in order to obtain complementary data to facilitate decision-making in treating patients properly [38, 39]. Measures have been implemented to gauge satisfaction among patients who participate in clinical trials during the preliminary stages of developing a new drug. This information is intended to serve as a supplemental resource when determining the optimal dosage for that drug in subsequent clinical trials or under real-world healthcare contexts [11, 42].

The instrument was developed based on the known structure of the generic SATMED-Q [23, 43]. Nevertheless, the panel of experts deemed it necessary to unfold the effectiveness dimension into two separate subscales: effectiveness in crisis and effectiveness in prevention. Additionally, a provisional new dimension measuring emotions towards treatment was incorporated to augment the original model and to broaden the scope of experiences undergone by the patient (in accordance with the general framework of patient-reported experience measurements). The initial structure comprised eight related dimensions, each measured by 35 items. The inter-rater valuations demonstrated that the proposed items were capable of defining each of the dimensions and concepts, which were found to be well isolated. The item reduction process demonstrated that all dimensions were well formed, unidimensional, and reliable in isolation. This suggested the possibility of retaining initial reliability while reducing the number of items to a minimum of three, as necessary for local identification. Two exceptions were identified. The convenience dimension did not attain the anticipated level of reliability; nevertheless, the decision was made to retain the dimension to align with the original measurement model. Furthermore, the broad array of treatment options available suggests that a multitude of issues pertaining to medication regimens and administration routes may be contributing to the variability in dimensional internal consistency. Nonetheless, convenience was theorized to be a potential source of dissatisfaction that necessitated measurement. Moreover, patient emotions did not demonstrate the desired level of reliability. Consequently, it was decided to eliminate the patient emotions variable from the model, given that general satisfaction was also measured by the model.

The 22-item reduced form showed good fit to the theoretical model in a fresh sample, and reliability values were retained. An assessment of internal consistency and temporal stability confirmed that the questionnaire’s total and subscale scores met or surpassed the recommended reliability standards [44]. Subscale correlations with concurrent instruments showed that higher correlations were obtained with symptom impact as measured with the HIT-6 concurrent measurement, and to a minor extent with HRQoL divergent measurements. It should be noted that scores for satisfaction with medical care were overly high and biased, perhaps because patients desiring to participate in the study were suffering from a chronic condition and felt confident with their practitioners. This later result should be tested in more detail with newly diagnosed patients. At the time the study was conducted, the frequency of pain days did not match the corresponding initial diagnosis, since patients could have fewer headache days due to their treatments. That is why a high percentage of patients with chronic migraine diagnosis had less than 15 monthly headache days at the time of the study. Although patient migraine severities varied at the time of measurement.

The use of this new instrument provides an opportunity to assess patient satisfaction with their treatment in the absence of the practitioner and prior to face-to-face interaction. It should offer the patient more opportunities to express their drawbacks regarding current treatment and function as an alarm signal. It is anticipated that values for dimensions such as medical care, which have demonstrated a high degree of importance, will be particularly sensitive to any decline in these values. The reliability of the questionnaire was assessed by examining its internal consistency and test-retest reliability. The results indicated that both the total composite score and the subscales demonstrated values above the established minimum standards [44]. Furthermore, it enables differentiation between the impact of acute symptoms and preventive treatments, while concurrently addressing the issue of undesired side effects, which in some cases may signify treatment overload. The results from the LCA demonstrate that the utilized questionnaire enables the identification of patient groups with disparate scores, which may possess clinical significance. Despite the modest sample size, the study revealed a range of satisfaction levels. For instance, cluster number 3, which comprises 21% of the patient population, has been identified as a group exhibiting elevated levels of satisfaction across all dimensions. This cluster, therefore, can be understood as comprising patients who have expressed the highest levels of satisfaction. Convenience is the dimension with lower scores for this cluster, which is only significantly different from the average scores in cluster 4 (the less satisfied cluster, *p* = 0.03). On the opposite extreme, satisfaction with medical care is the one with higher average scores for all clusters and it is not able to distinguish between clusters. This does not mean that this dimension is unable to identify unsatisfied patients, as it does differentiate between patients in the higher quartile and those in the lower quartile (*p* < 0.001). We can see that satisfaction with medical care is high for most patients (and the groups they are in). In fact, this was expected since patients have the opportunity to follow treatment with a different practitioner when they are not satisfied. Nonetheless, it is possible to differentiate patients based on their level of satisfaction, and not all patients are equally satisfied with their medical care (perhaps because they are not satisfied with other dimensions of treatment). It is also possible to see that the group with lower scores (cluster 4, *n* = 19) is the one with a higher proportion of chronic patients (76%), and it is the one with a higher score in HIT-6 (*M* = 72 over 100). In those aspects, it is similar to cluster 2, but cluster 3 has a higher proportion of patients with undesired side-effects (55%), and very low scores in the two dimensions of effectiveness (crisis = 0.23; prevention = 0.15) and in the impact on daily activities (inverted = 0.25). We can see that MISAT-Q may have meaning from a clinical perspective and does reflect the patient situation during treatment.

One limitation of this study is that it involves a crosssectional design and is not capable of examining casual influences of low treatment satisfaction on clinically relevant outcomes. In addition, responsiveness to change of this questionnaire was not examined given the crosssectional study design. Prospective studies are planned to address this issue. Drug interactions and comorbidities have not been studied, and the current design does not enable such inquiries. The study did not specify whether potential confounding variables, such as psychiatric comorbidities (e.g., depression, anxiety), migraine subtype, or concomitant treatments, were controlled for or analyzed, all of which could influence patient satisfaction and treatment perceptions. It also did not include a comparison with other established migraine-specific satisfaction PREMs, precisely because such instruments are currently lacking, which makes it challenging to position the MISAT-Q in relation to existing tools. Further studies involving other diseases and different drugs are needed to confirm the findings. A final limitation of the MISAT-Q is that the primary validation included patient samples obtained in Headache Units (with a high percentage of chronic and refractory patients), with no stratification including patients consecutively. Consequently, the validation of this instrument in international settings and/or in primary care should be tested taking into account the possible drawbacks mentioned.

## Conclusion

The MISAT-Q is a novel PREM to assess treatment satisfaction, specific to migraine patients. The findings in this study suggest that the instrument has good acceptability as well as satisfactory psychometric properties, including validity and reliability of the subscales and composite score. The findings support the use of the MISAT-Q as a measure of treatment satisfaction with medication both in daily medical practice and in clinical research. Moreover, the MISAT-Q instrument may contribute to our understanding of patient medicationrelated decisions and behaviors, thus proving the MISAT-Q is both an important determinant and outcome of effective clinical care and treatment adherence.

## Electronic supplementary material

Below is the link to the electronic supplementary material.

Supplementary Material 1

## Data Availability

The data that support the findings of this study are available from Universidad Autónoma de Madrid but restrictions apply to the availability of these data, which were used under license for the current study, and are not publicly available. Data are however available from the authors upon reasonable request and with permission of Universidad Autónoma de Madrid.

## Abbreviations

CFI: Comparative fit index
CFA: Confirmatory factor analysis
EFA: Exploratory factor analysis
HIT-6: Headache Impact Test- six items
HRQoL: Health-related quality of life
ICHD: International Classification of Headache Disorders
HIS: International Headache Society
LCA: Latent Class Analysis
MHD: Migraine headache days
MIDAS: Migraine Disability Assessment Scale
MSQ: Migraine-Specific Quality of Life Questionnaire
MTAQ: Migraine Therapy Assessment Questionnaire
MTSM: Migraine Treatment Satisfaction Measure
PGI: Patient Global Impression scale
PPM-Q: Patient Perception of Migraine Questionnaire
PPMQ-R: Patient Perception of Migraine Questionnaire-Revised
PREM: Patient-Reported-Experience Measure
PRO: Patient-Reported-Output
PROM: Patient-Reported-Outcome Measure
RMSEA: Root mean square error of approximation
SATMED-Q: Satisfaction with Treatment with Medicines Questionnaire
SEM: Standard Error of Measurement
TLI: Tucker-Lewis index
