# Supplementary material for: Development and validation of a patient-reported-experience measure to ascertain treatment satisfaction in migraine: the MISAT-Q questionnaire
Source: J Patient Rep Outcomes. 2025 Dec 15;10:10. doi: 10.1186/s41687-025-00979-x (PMC12819897; doi:10.1186/s41687-025-00979-x)
Supplement: Supplementary file 1 — Supplementary Material 1 [file 41687_2025_979_MOESM1_ESM.docx]

**Development and validation of a Patient-Reported-Experience Measure to ascertain Treatment Satisfaction in Migraine: The MISAT‑Q questionnaire**

**Ana B. Gago-Veiga^1,2^, Nuria González-García^2,3^, Javier Díaz-de-Terán^2,4^, Patricia Heredia-Rodríguez^1,5^, Beatriz Armada-Peláez^6^, Carlota Moya-Alarcón^7^, Javier Soto-Álvarez^8^, Javier Rejas-Gutiérrez^9^, Miguel Á. Ruiz-Díaz^9,10^**

^1^Headache Unit, Hospital Universitario La Princesa, Madrid, Spain; ^2^Group for the Study of Headache, Spanish Society of Neurology (GECSEN), Spain; ^3^Headache Unit, Hospital Clínico San Carlos, Madrid, Spain; ^4^Headache Unit, Hospital Universitario La Paz, Madrid, Spain; ^5^Group for the Study of Headache, Spanish Society of Nursing Neurology (GECSEDENE), Spain; ^6^Medical Department, Pfizer S.L.U., Alcobendas, Madrid, Spain; ^7^Health Outcomes Research Department, Pfizer S.L.U., Alcobendas, Madrid, Spain; ^8^MEVAFARMA, Universidad Carlos III, Madrid, Spain; ^9^EACCOS Research Group, Universidad Autónoma de Madrid, Spain; ^10^School of Psychology, Universidad Autónoma de Madrid, Spain.

**SUPPLEMENTARY INFORMATION**

**Sample size justification**

Four different samples were used: 1) *pilot-content selection sample*: comprised of 23 randomly recruited patients; 2) *reduction sample*: 158 patients under migraine treatment; and 3) *validation sample*: 209 patients selected considering the same criteria as in the reduction sample; and 4) *retest sample*: a subgroup of 32 patients was selected from those in the validation sample. No stratification criteria were deemed necessary in order to require a stratified sampling procedure. The size of the pilot sample was considered enough to evaluate feasibility, pertinence, and content suitability of items, and to determine whether the items were clearly understood by the patients. The size of the reduction sample was determined based on a ratio subjects/variable not less than 4/1, and at least 100 patients. Considering the number of items of the first version of the questionnaire, and moreover, considering that some subjects could give non‑evaluable answers, a minimum of 140 patients was considered advisable. Patient selection was random and sequential, until the indicated subject quotas by center were covered. To apply factor analysis, the validation sample needed 88 patients. This number was based on the same criteria as the final questionnaire, which had 22 questions in total after the item reduction stage. However, the sample size was over‑dimensioned to allow stable estimates of the factor structure requiring a minimum of 150 patients with complete answers. The reduction sample finally comprised 158 patients. Although a similar patient quota was assigned to each of the participating centers, recruitment was carried out competitively among the centers to accelerate the patient recruitment process. The validation sample comprised 228 patients, but only 209 patients had complete information. Nonetheless, was considered sufficient to test validity attributes of final version of MISAT-Q (see references 21 and 22 in main manuscript).

**Item Reduction**

The revised questionnaire incorporating the contributions of the pilot sample was in turn administered to the reduction sample by an anonymized electronic format. The information obtained from this sample was then used for the following purposes: (1) to check adjustment of the patient responses to the structure (dimensions or subscales) proposed by the group of experts; (2) to assess the metric properties of the items; and (3) to reduce the number of questions to a maximum of three per dimension.

Reduction of the questionnaire and determination of the underlying dimensions were carried out via a sequence of exploratory factor analyses, based on the assessment of internal consistency. Two extraction methods were used: principal components and principal axes (while maximum likelihood was suspected to entail estimation problems due to the expected non-normal distribution of scores); and two rotation methods: varimax (orthogonal) and oblimin (oblique). Heuristics for determining the optimum number of factors comprised the Kaiser K1 rule, scree test, the percentage of variance accounted for, and the magnitude of the eigenvalues after rotation. A number of decision rules were used, due to the tendency of all of them to either underestimate or overestimate the correct number of factors in different contexts. Internal consistency was evaluated by means of Cronbach’s alpha reliability coefficient, and the change in alpha coefficient after deleting each item from the scale (see references 30 to 33 in main manuscript).

In this reduction of the length of the questionnaire and analysis of dimensionality, we adopted the proposals by Gorusch and Russell (see references 34 and 35 in main manuscript). Firstly, items with a clear floor or ceiling effect (i.e., items with more than 70% of answers concentrated in the first or last answer category) were identified. Secondly, an exploratory factor analysis was made with the 35 items composing the scale, to determine the number of underlying factors or dimensions (subscales). Lastly, isolated subscales were analyzed for unidimensionality (factor analysis) and internal consistency (Cronbach’s alpha coefficient). Items with communalities less or equal to 0.2 were candidates to be eliminated from the preliminary version of the questionnaire. Also, items were eliminated when Cronbach’s alpha coefficient improved after item deletion.

**Additional questionnaires**

*Headache Impact Scale–6 items (HIT-6):* Developed to measure a wide spectrum of factors contributing to the burden of headache, it has demonstrated utility for generating quantitative and pertinent information on the impact of headache. It is frequently used in clinical practice to assess severity. This questionnaire was included as a convergent measure of headache perceived severity and is expected to correlate (negatively) with our instrument.

*Health related quality of life (HRQoL) state EuroQoL (EQ-5D-3L);* The EQ-5D is a concise, generic measure of self-reported health which is accompanied by weights reflecting the relative importance to people of different types of health problems. This instrument was included being a widely used generic measure of disease impact, and is expected to correlate with our instrument less than the HIT-6.

*HRQoL Visual Analogic Scale (VAS):* A single-item generic measure of present quality of life state which usually accompanies the EQ-5D. This is a second generic measure, with less reliability.

*Patient Global Impression scale (PGI):* Patient opinion on his/her health improvement in the last 30 days. This instrument was included to assess perceived changes over time. The Patient Global Impression of Improvement (PGI-I) is a global index that may be used to rate the response of a condition to a therapy (transition scale).  It is a simple, direct, easy to use scale that is intuitively understandable to clinicians. The PGI-I is a transition scale that is a single question asking the patient to rate their urinary tract condition now, as compared with how it was prior to before beginning treatment on a scale from 1.  Very much better to 7.  Very much worse.

**Psychometric properties of the final version**

The abridged or final version of the questionnaire was included in a case report form (CRF), together with clinical information of relevance for the patient, sociodemographic information, EQ-5D-3L and HIT-6. The CRF was answered by patients from the validation sample using an electronic format with an anonymization code. The data obtained from this sample were then used for the following: (1) to ratify the structure of the abridged questionnaire, (2) to assess the metric properties of the questionnaire; and (3) to elaborate norms for the Spanish population. The following metric properties of the final questionnaire were studied: (1) *feasibility:* administration time, floor and ceiling effects, percentage of missing values in each item; (2) *reliability*: internal consistency, evaluated by means of Cronbach’s alpha coefficient and the Pearson correlation coefficient between items and between each item and the total composite score; (3) test‑retest (temporal stability), evaluated by correlating two administrations of the questionnaire based on the Pearson correlation coefficient and intraclass correlation coefficient; (4) *construct validity*: the structure in dimensions of the answers gathered with the final questionnaire was established by confirmatory factor analysis; (5) *concurrent validity*: correlations between the HIT-6 scale and the VAS scores were assessed; (6) *divergent validity*: correlation with EQ-5D-3L scores were expected to be lower than between questionnaire dimensions; and (7) *discriminant validity*: an analysis was made of the capability of each subscale to discriminate between the 25% of subjects with the lowest scores and the 25% with the highest scores (established from the total composite scale scores). Comparisons were also made between groups known to behave differently based on PGI scores grouped in 3 levels: very much worse, much worse=worse; somewhat worse, no change, somewhat better=no change; much better, very much better=better.

We briefly the following main psychometric properties being tested. (1) *Feasibility:* face value (good acceptance to answer the questionnaire), administration time (below 10 minutes), floor and ceiling effects (below 40% in any extreme response category), percentage of missing values in each item (below 20%). (2) *Reliability*: internal consistency or mutual interrelation between items, evaluated by means of Cronbach’s alpha coefficient (average correlation between items; above 0,80) and Pearson correlation between each item and the total composite score (discarding those below Cronbach’s alpha). (3) *Test‑retest reliability*: temporal stability between measurements to the same patients, assessed by correlating two administrations of the questionnaire separated by a small span of time, computing Pearson correlation coefficient and intraclass correlation coefficient. (4) *Construct validity*: the structure in dimensions of the answers gathered from patients, established by Confirmatory Factor Analysis (*structural validity*), and relying on the results obtained from Exploratory Factor Analysis for item reduction. (5) *Concurrent validity*: expecting high correlations with other questionnaires measuring similar concepts/constructs; (6) *Divergent validity*: expecting low correlations with other questionnaires and dimensions measuring different conceptual concepts. (7) *Discriminant validity*: capability of each dimension to discriminate between patients with highest and those with lowest scores; and capability to distinguish between patients who have experienced a change from those not experiencing change.

Figure S1. Age distribution of validity sample with frequency histogram.


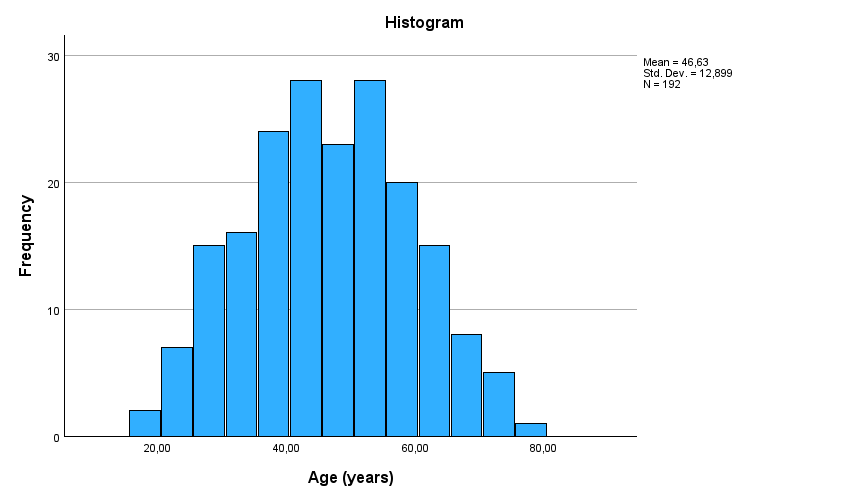


Table S1. Number of migraine episodes in 4 weeks in reduction and validity samples.

|  | Reduction Sample | | Validation Sample | |
| --- | --- | --- | --- | --- |
|  | Frequency | Percent | Frequency | Percent |
| 0 | 1 | 0.9 | 0 | .0 |
| 1 | 4 | 3.4 | 3 | 1.6 |
| 2 | 8 | 6.9 | 10 | 5.2 |
| 3 | 7 | 6.0 | 18 | 9.4 |
| 4 | 5 | 4.3 | 20 | 10.4 |
| 5 | 4 | 3.4 | 8 | 4.2 |
| 6 | 12 | 10.3 | 24 | 12.5 |
| 7 | 4 | 3.4 | 5 | 2.6 |
| 8 | 6 | 5.2 | 3 | 1.6 |
| 9 | 11 | 9.5 | 9 | 4.7 |
| 10 | 1 | 0.5 | 12 | 6.3 |
| 11 | 7 | 6.0 | 9 | 4.7 |
| 12 | 9 | 7.8 | 7 | 3.6 |
| 13 | 1 | 0.9 | 1 | .5 |
| 14 | 2 | 1.7 | 2 | 1.0 |
| 15 | 4 | 3.4 | 10 | 5.2 |
| 16 | 6 | 5.2 | 6 | 3.1 |
| 17 | 0 | .0 | 1 | .5 |
| 18 | 5 | 4.3 | 2 | 1.0 |
| 19 | 0 | .0 | 2 | 1.0 |
| 20 | 5 | 4.3 | 12 | 6.3 |
| 21 | 1 | .9 | 0 | .0 |
| 22 | 1 | .9 | 0 | .0 |
| 23 | 0 | .0 | 0 | .0 |
| 24 | 1 | .9 | 0 | .0 |
| 25 | 2 | 1.7 | 5 | 2.6 |
| 26 | 1 | .9 | 0 | .0 |
| 27 | 0 | .0 | 0 | .0 |
| 28 | 0 | .0 | 2 | 1.0 |
| 29 | 0 | .0 | 2 | 1.0 |
| 30 | 8 | 6.9 | 19 | 9.9 |
| Total | 116 | 100.0 | 192 | 100.0 |

Table S2. Percentage of missing values by dimension. Reduction Sample.

|  | Number of missing values | | | | | |
| --- | --- | --- | --- | --- | --- | --- |
|  | 0 | 1 | 2 | 3 | 4 | 5 |
| Undesirable side effects | 99.3 | 0.7 | 0.0 | 0.0 | 0.0 | 0.0 |
| Crisis effectiveness | 97.3 | 1.4 | 1.4 | 0.0 | 0.0 | 0.0 |
| Prevention effectiveness | 98.6 | 0.7 | 0.0 | 0.7 | 0.0 | 0.0 |
| Convenience of use | 89.9 | 7.4 | 0.7 | 0.7 | 0.0 | 1.4 |
| Impact on daily activities | 89.2 | 8.8 | 1.4 | 0.0 | 0.7 | 0.0 |
| Medical care | 91.9 | 6.8 | 1.4 | 0.0 | 0.0 | 0.0 |
| Emotions | 90.5 | 6.8 | 1.4 | 1.4 | 0.0 | 0.0 |
| Global satisfaction | 94.6 | 4.1 | 1.4 | 0.0 | 0.0 | 0.0 |

Table S3. MISAT-Q dimension and total score descriptive statistics

|  | N | Minimum | Maximum | Mean | Std. Deviation |
| --- | --- | --- | --- | --- | --- |
| Undesirable side effects | 169 | ,00 | 100,00 | 78,1065 | 30,42574 |
| Crisis effectiveness | 169 | ,00 | 100,00 | 67,7515 | 26,65749 |
| Prevention effectiveness | 169 | ,00 | 100,00 | 70,8580 | 30,67846 |
| Convenience of use | 165 | ,00 | 100,00 | 68,1566 | 24,09855 |
| Impact on daily activities | 167 | ,00 | 100,00 | 68,4132 | 28,85421 |
| Medical care | 167 | 16,67 | 100,00 | 90,1697 | 16,07393 |
| Global satisfaction | 168 | 16,67 | 100,00 | 81,7708 | 21,29326 |
| Total composite score | 169 | 29,76 | 100,00 | 75,0142 | 17,37924 |

Table S4. Average MISAT-Q total score by PGI severity group. Validation Sample.

| PGI severity | N | MISAT-Q | | Pairwise differences | |
| --- | --- | --- | --- | --- | --- |
|  |  | Average | SD | No-change | Worse |
| Better | 48 | 84.50 | 16.9 | 10.99 (p<0.001) | 24.72 (p<0.001) |
| No change | 142 | 73.50 | 17.4 |  | 13.72 (p=0.006) |
| Worse | 14 | 59.78 | 14.0 |  |  |

**Latent Class Analysis**

Latent Class Analysis generated four clusters with profiles that resembled a progressive drop in satisfaction in all seven dimensions, in the following decreasing order of satisfaction: cluster 3 (21%), cluster 1 (43%), cluster 2 (23%) and cluster 4 (12%). Clusters are labeled by descending number of cases contained in the group.

Table S5. Number of cases in each Latent class group.

|  | | Frequency | Percent | Cumulative Percent |
| --- | --- | --- | --- | --- |
|  | 1 | 73 | 43,2 | 43,2 |
|  | 2 | 39 | 23,1 | 66,3 |
|  | 3 | 36 | 21,3 | 87,6 |
|  | 4 | 21 | 12,4 | 100,0 |
|  | Total | 169 | 100,0 |  |

The following tables display, for each dimension, average scores for each cluster (in the diagonal) and pairwise comparison between centroids (off-diagonal).

Table S6. Undesirable side effects average scores by cluster and cluster pairwise comparison significance

| Cluster | 1 | 2 | 3 | 4 |
| --- | --- | --- | --- | --- |
| 1 | 80.13 |  |  |  |
| 2 | .692 | 74.14 |  |  |
| 3 | .032 | .005 | 95.60 |  |
| 4 | <.001 | .004 | <0.001 | 48.41 |

Note. Off-diagonal: two-tail sig, diagonal: observed cluster averages,

Table S7. Crisis Effectiveness average scores by cluster and cluster pairwise comparison significance

| Cluster | 1 | 2 | 3 | 4 |
| --- | --- | --- | --- | --- |
| 1 | 72.72 |  |  |  |
| 2 | <.001 | 56.84 |  |  |
| 3 | <.001 | <.001 | 96.06 |  |
| 4 | <.001 | <.001 | <0.001 | 22.22 |

Note. Off-diagonal: two-tail sig, diagonal: observed cluster averages,

Table S8. Prevention Effectiveness average scores by cluster and cluster pairwise comparison significance

| Cluster | 1 | 2 | 3 | 4 |
| --- | --- | --- | --- | --- |
| 1 | 82.28 |  |  |  |
| 2 | <.001 | 55.13 |  |  |
| 3 | <.001 | <.001 | 97.56 |  |
| 4 | <.001 | <.001 | <0.001 | 14.58 |

Note. Off-diagonal: two-tail sig, diagonal: observed cluster averages,

Table S9. Convenience of Use average scores by cluster and cluster pairwise comparison significance

| Cluster | 1 | 2 | 3 | 4 |
| --- | --- | --- | --- | --- |
| 1 | 67.90 |  |  |  |
| 2 | .851 | 64.10 |  |  |
| 3 | .169 | .060 | 77.98 |  |
| 4 | .529 | .911 | 0.034 | 59.79 |

Note. Off-diagonal: two-tail sig, diagonal: observed cluster averages,

Table S10. Impact on daily activities average scores by cluster and cluster pairwise comparison significance

| Cluster | 1 | 2 | 3 | 4 |
| --- | --- | --- | --- | --- |
| 1 | 80.02 |  |  |  |
| 2 | <.001 | 44.41 |  |  |
| 3 | <.001 | <.001 | 97.14 |  |
| 4 | <.001 | <.001 | <0.001 | 23.61 |

Note. Off-diagonal: two-tail sig, diagonal: observed cluster averages,

Table S11. Medical Care average scores by cluster and cluster pairwise comparison significance

| Cluster | 1 | 2 | 3 | 4 |
| --- | --- | --- | --- | --- |
| 1 | 91.84 |  |  |  |
| 2 | .212 | 85.59 |  |  |
| 3 | .945 | .138 | 93.63 |  |
| 4 | .530 | .997 | .363 | 86.51 |

Note. Off-diagonal: two-tail sig, diagonal: observed cluster averages,

Table S12. Global satisfaction average scores by cluster and cluster pairwise comparison significance

| Cluster | 1 | 2 | 3 | 4 |
| --- | --- | --- | --- | --- |
| 1 | 90.05 |  |  |  |
| 2 | <.001 | 69.77 |  |  |
| 3 | .007 | <.001 | 98.84 |  |
| 4 | <.001 | <.001 | <0.001 | 46.43 |

Note. Off-diagonal: two-tail sig, diagonal: observed cluster averages,

Table S13. MISAT-Q dimension score ANOVA by MISAT-Q total score quartile group.

| MISAT-Q dimension | ANOVA | | Linear Trend | |
| --- | --- | --- | --- | --- |
|  | F | Sig. | F | Sig |
| Adverse Events | 20.44 | <.001 | 58.54 | <.001 |
| Crisis Effectiveness | 70.99 | <.001 | 211.31 | <.001 |
| Prevention Effectiveness | 82.26 | <.001 | 224.22 | <.001 |
| Convenience of use | 7.59 | <.001 | 22.68 | <.001 |
| Impact on daily activities | 102.81 | <.001 | 295.51 | <.001 |
| Medical Care | 7.66 | <.001 | 17.90 | <.001 |
| General Satisfaction | 80.94 | <.001 | 222.50 | <.001 |

Table S14. Average scores in Medical care dimension score (0-1) by MISAT-Q total score quartile group.

| Dependent Variable: Medical Care | | | |
| --- | --- | --- | --- |
| Quartile Group of MISAT Total | Mean | Std. Deviation | N |
| Q1 | .8150 | .22651 | 41 |
| Q2 | .9207 | .11016 | 41 |
| Q3 | .8968 | .15295 | 42 |
| Q4 | .9709 | .07902 | 43 |
| Total | .9017 | .16074 | 167 |

Table S15. Multiple pairwise comparisons on scores in Medical care by MISAT-Q quartile group. Tukey Honestly Significant Difference.

| (I) Percentile Group of MISAT Total | (J) Percentile Group of MISAT Total | Mean Difference (I-J) | Std. Error | Sig. | 95% Confidence Interval | |
| --- | --- | --- | --- | --- | --- | --- |
|  |  |  |  |  | Lower Bound | Upper Bound |
| Q1 | Q2 | -.1057^*^ | .03354 | .010 | -.1928 | -.0186 |
|  | Q3 | -.0818 | .03334 | .071 | -.1683 | .0048 |
|  | Q4 | -.1559^*^ | .03315 | <.001 | -.2419 | -.0698 |
| Q2 | Q1 | .1057^*^ | .03354 | .010 | .0186 | .1928 |
|  | Q3 | .0239 | .03334 | .890 | -.0626 | .1104 |
|  | Q4 | -.0502 | .03315 | .431 | -.1362 | .0358 |
| Q3 | Q1 | .0818 | .03334 | .071 | -.0048 | .1683 |
|  | Q2 | -.0239 | .03334 | .890 | -.1104 | .0626 |
|  | Q4 | -.0741 | .03295 | .115 | -.1596 | .0114 |
| Q4 | Q1 | .1559^*^ | .03315 | <.001 | .0698 | .2419 |
|  | Q2 | .0502 | .03315 | .431 | -.0358 | .1362 |
|  | Q3 | .0741 | .03295 | .115 | -.0114 | .1596 |
| Based on observed means.  The error term is Mean Square (Error) = .023. | | | | | | |
| *. The mean difference is significant at the .05 level. | | | | | | |

**COSMIN Reporting guideline for studies on measurement properties of patient reported outcome measures**

**Version August 2021**

Joel J Gagnier, Jianyu Lai, Lidwine B Mokkink, Caroline B Terwee. COSMIN reporting guideline for studies on measurement properties of patient-reported outcome measures. Qual Life Res. 2021 Aug; 30(8):2197-2218. [doi: 10.1007/s11136-021-02822-4](https://link.springer.com/content/pdf/10.1007/s11136-021-02822-4.pdf).

| **General Reporting recommendations relevant for all studies on measurement properties** | | |  |
| --- | --- | --- | --- |
| **Item Number** | **Item Name** | **Item Description** |  |
| **Report section: Title** | |  |  |
| T1 | Patient Reported Outcome Measure (PROM) | Treatment Satisfaction in Migraine: The MISAT‑Q questionnaire |  |
| T2 | Measurement Property (MP) | Treatment satisfaction in patients with migraine |  |
| T3 | Study sample | Patients with migraine receiving treatment |  |
| **Report section: Abstract** | |  |  |
| A1 | PROM | The aim was to develop and validate a novel specific self-reported patient-reported-outcomes measurement (PROM) in Spanish for Spain to ascertain satisfaction with migraine treatment; the MISAT-Q questionnaire. |  |
| A2 | Measurement Property | Feasibility, reliability and validity (content, discriminant, construct, and concurrent) of a satisfaction with treatment measure |  |
| A3 | Design | A multicenter, cross‑sectional, non-interventional study was conducted between June 2023 and March 2024 in headache units in Spain. Measures for concept validity included Patient Global Impression of change (PGI), Headache Impact Scale-6 items (HIT-6), and Health-Related Quality-of-life (EQ-5D-3L). Feasibility, reliability and validity (content, discriminant, construct, and concurrent) were assessed. |  |
| A4 | Sample | Patients with migraine receiving treatment of their headache were included in the study in headache units in Spain. Three samples were used: a) 23 patients to assess feasibility/perti­nence of items; b) 158 patients for item reduction; and c) 209 patients for psychometric properties assess­ment of PROM. |  |
| A5 | Methods | Feasibility, reliability and validity (content, discriminant, construct, and concurrent) were assessed. |  |
| A6 | Results | Factor analysis item reduction resulted in a 22‑item questionnaire with 7 dimensions: undesi­ra­ble side effects, treatment effectiveness in crisis and in prevention, convenience-of-use, impact on daily activities, medical care, and global satisfaction, supported with confirmatory factor analysis: CFI=0.983; TLI=0.980; χ^2^/df=1.808; RMSEA=0.075. Reliability was high: Cronbach’s alpha =0.90 and intraclass-correlation coefficient =0.95. Dimensions of questionnaire showed signifi­cant and moderate correlations with the overall score (0.58-0.68, p<0.001). Concurrent validity with HIT-6 showed correlations ranging from -0.19 to -0.48 (*p*<0.01 in all cases, except medical care) and with EQ-5D-3L (correlations from 0.17 to 0.39, *p*<0.05 in all cases, except medical care). Differences in satisfaction were found according with migraine severity (F=6.73, p=0.002), as well as in HIT-6 scores (F=8.20, p<0.002). MISAT-Q discriminated overall satisfaction between patients with worse, no change and better change with treatment (F=15.85; p<0.001). |  |
| A7 | Discussion/Conclusions | The MISAT‑Q questionnaire is a feasible, reliable and valid measure of migraine treatment satisfaction in Spanish. Responsiveness attribute needs to be further explored. This novel PROM may facilitate clinicians when making health decision in the treatment management of patients with migraine. |  |
| **Report section: Introduction** | |  |  |
| I1 | Name and describe the PROM of interest | There is still a need of instruments for assessing satisfaction with the migraine treatments in a holistic way, covering peculiarities of migraine treatment as much as possible, particularly being able to differentiate the most recent migraine treatments and medical attention received, all the above known to have an impact on patient satisfaction. The aim of this study was to develop and validate a novel specific multidimensional self-reported patient-reported-outcomes measurement (PROM) to ascertain satisfaction with migraine treatment in Spanish for Spain; the MISAT-Q questionnaire.  More information appears in the methods section in greater detail. |  |
| I2 | Target population | Tools assessing patient´s satisfaction with migraine treatments such as the Patient Perception of Migraine Questionnaire (PPM-Q), its revised version (PPMQ-R) or the Migraine Treatment Satisfaction Measure (MTSM) are available. Even such instruments are reliable and accurate tool to measure patient satisfaction with various migraine treatment options or can provide valuable information with different aspects of migraine impact in patient´s daily living, it is important to note that mentioned PROMs are actually not capturing the whole spectrum of patient´s satisfaction with therapy; the MTSM is not a measure of treatment effectiveness or efficacy, the PPMQ-R evaluates the level of patient satisfaction with treatment for acute migraine attacks in five areas: adverse effect discomfort, ease of use, cost, functionality, and efficacy, excluding prevention, medical care or global satisfaction. Also, even providing valuable information on treatment effectiveness, such tools may not capture all aspects of a patient’s satisfaction with their treatment, advising to use several questionnaires in conjunction to obtain a more comprehensive evaluation of the treatment. |  |
| I3 | Citation for the original development of the PROM | Not applicable. The study describes the development of a novel PROM for the first time. |  |
| I4 | State of Knowledge & Rationale | See Introduction section, pages 5 and 6 of manuscript. |  |
| I5 | Definitions | See page 4 with abbreviations and supplementary information |  |
| I6 | Objectives and Hypotheses | The aim of this study was to develop and validate a novel specific multidimensional self-reported patient-reported-outcomes measurement (PROM) to ascertain satisfaction with migraine treatment in Spanish for Spain; the MISAT-Q questionnaire. |  |
| **Report section: General Methods** | |  |  |
| GM1 | Study Design | A multicenter, cross‑sectional, non-interventional study was conducted between June 2023 and March 2024 at three Headache Units in Madrid (Spain): Hospital Universitario de La Princesa, Hospital Clínico San Carlos and Hospital Universitario La Paz. |  |
| GM2 | Participants | Patients were sequentially selected among those who met the following selection criteria: outpatients with ≥18 years of age, diagnosed with episodic (<15 monthly headache days, MHD) or chronic migraine (≥15 MHD during more than three consecutive months) according to International Classification of Headache Disorders (ICHD-3), initiating or following acute or preventive migraine treatment at the time of selection, able to understand and answer the health questionnaires included in the study (Spanish versions, see supplemental information with a brief description of questionnaires used), and willing to sign the informed consent form. Patients participating in any clinical trial about migraine treatment were excluded. |  |
| GM3 | PROM administration | See section methods, pages 7 and 8 of main manuscript and supplemental information. |  |
| GM4 | Data collection procedures | See section methods, pages 7 and 8 of main manuscript and supplemental information. |  |
| GM5 | Power/sample size calculation | See section methods, page 7 of main manuscript and supplemental information with sample size calculation in detail. |  |
| GM6 | Statistical analyses | See section methods, pages 8 to 10 with questionnaire development description including test to confirm MPs and page 10 with statistical analysis section. |  |
| GM7 | Missing data | Not applicable due to the lower rate of missing data observed in the study. |  |
| GM8 | Post hoc analysis | Not applicable. |  |
| **Report section: General Results** | |  |  |
| GR1 | Missing data | Table 1 reports sociodemographic and clinical information for the reduction and validation samples. Missing data was low and completely at random, with data lacking in less than 10% of patients enrolled in the study. Therefore, imputation of missing data was not applied. |  |
| GR2 | Participant/patient Characteristics | See table 1. |  |
| GR3 | Sample size | See section GM5 of this guideline and section results, pages 11 and 12. |  |
| **Report section: Discussion** | |  |  |
| D1 | MP evidence | See pages 15, 16 and 17 and results section including tables 2 to 6 and figure 1. | |
| D2 | Practical relevance | See page 17. | |
| D3 | Strengths and limitations | See pages 16 and 17. | |
| D4 | Generalizability | Not applicable. The PROM included here is specific of migraine treatment. Therefore, generalizability to other populations given the sample studied if not possible out of other migraine samples. | |
| D5 | Instrument changes | Not applicable as MP assessed are acceptable. The only MP limitation is that responsiveness of the PROM needs to be assessed in a longitudinal additional study. | |
| D6 | Future Research | In addition to commented in box D5, drug interactions and comorbidities have not been studied, as the present design does not allow such inquiries. Further studies involving other diseases and different drugs are needed to confirm the findings. A final limitation of the MISAT-Q is that the primary validation included patient samples obtained in Headache Units (with a high percentage of chronic and refractory patients), with absence of stratification including patients consecutively; consequently, the validation of this instrument in international settings and/or in primary care should be tested taking into account some mentioned possible drawbacks. | |
| **Report section: Conclusions** | |  | |
| C1 | Conclusions | See page 18 | |
| **Report section: Other information** | |  | |
| O1 | Conflict of Interest | See pages 19 and 20 | |

| **Specific Reporting recommendations for studies on Content Validity** | | |
| --- | --- | --- |
| **Item Number** | **Item Name** | **Item Description** |
| CV1 | Relevance | See section Methods page 8  The questionnaire development process began with the selection of a panel of experts composed by three neurologists, a nurse specialized in headache, an expert patient, three health-outcomes-research specialists, and a methodologist, who supervised all phases of questionnaire development and validation. |
| CV2 | Comprehensiveness | See section Methods page 8  The panel of experts generated an initial series of questions in Spanish (Spain) relating to the following aspects: undesirable side effects, acute effectiveness, prevention effectiveness, global satisfaction, convenience of use, expectations, available clinical options, recommendation disposition, adherence, satisfaction with medical care, impact on daily life, emotions, and beliefs about treatments. Content validation implied thirteen migraine content-specialists (8 neurologists, 2 nurses, 1 psychologist, 2 health-outcomes-research specialists) valued each one of the proposed items in all dimensions measured and defined to scaffold the questionnaire (1=measured, 0=unsure, -1=not measured). |
| CV3 | Comprehensibility | See section Methods page 8  Items were worded in affirmative phrasing, and were designed to ensure that they referred to a single concept, and intending to be easy to answer. Possible answers were scored using a 5-point Likert‑type scale: 0 = “No, not at all”; 1 = “A little bit”; 2 = “Neither a lot, nor a little”; 3 = “Quite a lot”; 4 = “Yes, very much”. Complete agreement between content specialists was required, resulting in a preliminary version of questionnaire with 35 items and eight dimensions. This version was self-patient administered to the pilot-content selection sample, for assessment pertinence of possible items, missing contents, concept convenience, wording adequacy, and relevant concepts which might be missing or any difficulty found in answering the items or the presentation format. |
| CV4 | Relevance results | See section Methods pages 8 and 9 describing the process for item reduction and validation phase of final version of questionnaire |
| CV5 | Response options and recall period | Not applicable |
| CV6 | Comprehensiveness results | See section Methods pages 8 and 9 describing the process for item reduction and validation phase of final version of questionnaire |
| CV7 | Comprehensibility results | See section Methods pages 8 and 9 describing the process for item reduction and validation phase of final version of questionnaire |

| **Specific Reporting recommendations for studies on Structural Validity** | | |  |
| --- | --- | --- | --- |
| **Item Number** | **Item Name** | **Item Description** |  |
| SV1 | Factor Analyses: Classical Test Theory (CTT) PROMs | MISAT-Q was developed following classical test theory.  Section methods and results, pages 8 and 9 report details of both EFA and CFA applied. Results section, pages 12 and 13 and figure 1 report results of both EFA and CFA. EFA was used to explore the structure of the questionnaire (first version after item reduction). CFA was applied to confirm the dimensional structure of final version. Statistical analysis section on page 10 and supplemental information provides additional information regarding the use of EFA and CFA, including exploratory or confirmatory factor analyses, the model used and cut-off points for good model fit, including factor loadings of best-fitting model. |  |
| SV2 | Item Response Theory (IRT) analyses | Not applicable | |

| **Specific Reporting recommendations for studies on Internal Consistency** | | |
| --- | --- | --- |
| **Item Number** | **Item Name** | **Item Description** |
| IC1 | Unit of measurement | See page 9 in method section, and page 13 in results section. See also table 4 including internal consistency data. |
| IC2 | Continuous scores | See page 9 in method section, and page 13 in results section. See also table 4 including internal consistency data. |
| IC3 | Dichotomous scores | Not applicable. |

| **Specific Reporting recommendations for studies on Cross-Cultural Validity\Measurement Invariance** | | |
| --- | --- | --- |
| **Item Number** | **Item Name** | **Item Description** |
| CCV1 | Comparator Group(s) | Not applicable. The manuscript reports the development process of a novel PROM for the first time. |
| CCV2 | Factor Analyses: Classical Test Theory (CTT) PROMs | Not applicable. The manuscript reports the development process of a novel PROM for the first time. |
| CCV3 | Item Response Theory (IRT) analyses | Not applicable. The manuscript reports the development process of a novel PROM for the first time. |

| **Specific Reporting recommendations for studies on Reliability** | | |
| --- | --- | --- |
| **Item Number** | **Item Name** | **Item Description** |
| R1 | PROM Administrations | MISAT-Q was administered on two occasions to a stable subsample of 32 patients from those in the validation sample that answering the questionnaire one week apart approximately from the first administration. See page 7  The measurements were applied to the same samples using the same PROM. The process of administrating the measurements included that the patients completed the measurements. The setting was the headache units participating in the study. Also, the PROM was completed without knowledge of the previous scores of the administrations. |
| R2 | Statistical analyses | See page 9 |
| R3 | Methods to improve reliability | Not applicable. |

| **Specific Reporting recommendations for studies on Measurement Error** | | |
| --- | --- | --- |
| **Item Number** | **Item Name** | **Item Description** |
| ME1 | PROM administrations | All PROMs used in the study were self-administered to patients and only one time, except the subsample for temporal stability that was administered two times one week apart approximately with patient in stable status.  Questionnaires were completed by participant at home. In case of repeated administration, the PROM was completed without knowledge of the previous completion of the administrations. |
| ME2 | Statistical analyses | See Statistical section on pages 10 and 11 |

| **Specific Reporting recommendations for studies on Criterion Validity** | | |
| --- | --- | --- |
| **Item Number** | **Item Name** | **Item Description** |
| CriV1 | Criterion | Not applicable since there is no available a PROM of reference for satisfaction with migraine treatment available yet. |
| CriV2 | Continuous scores | Not applicable since there is no available a PROM of reference for satisfaction with migraine treatment available yet. |
| CriV3 | Categorical scores | Not applicable since there is no available a PROM of reference for satisfaction with migraine treatment available yet. |

| **Specific Reporting recommendations for studies on Hypotheses Testing for Construct Validity** | | |
| --- | --- | --- |
| **Item Number** | **Item Name** | **Item Description** |
| ConV1 | Comparator instrument(s) | See page 9 in methods section and supplemental information including a description of comparator instruments used to test construct validity, including related citations reporting the measurement properties of the comparator instruments. |
| ConV2 | Comparator Group(s) | See pages 9 and 10 in methods section and table 1. |
| ConV3 | Hypotheses | See pages 9 and 10 in methods section and supplemental information. |
| ConV4 | Statistical analyses | Statistical methods section report test used to test construct validity including testing structure and discriminant validity. |
| ConV5 | Results | See pages 13, 14 and 15 in results section and table 5. |

| **Specific Reporting recommendations for studies on Responsiveness** | | |
| --- | --- | --- |
| **Item Number** | **Item Name** | **Item Description** |
| Resp1 | Comparison Instrument(s) | Not applicable. This attribute is pending of measurement in additional studies. It is identified as a limitation in the manuscript. |
| Resp2 | Comparator Group(s) | Not applicable. This attribute is pending of measurement in additional studies. It is identified as a limitation in the manuscript. |
| Resp3 | Hypotheses | Not applicable. This attribute is pending of measurement in additional studies. It is identified as a limitation in the manuscript. |
| Resp4 | Measurement procedures | Not applicable. This attribute is pending of measurement in additional studies. It is identified as a limitation in the manuscript. |
| Resp5 | Interim period | Not applicable. This attribute is pending of measurement in additional studies. It is identified as a limitation in the manuscript. |
| Resp6 | Intervention/Exposure | Not applicable. This attribute is pending of measurement in additional studies. It is identified as a limitation in the manuscript. |
| Resp7 | Patients changed | Not applicable. This attribute is pending of measurement in additional studies. It is identified as a limitation in the manuscript. |
| Resp8 | Statistical analyses | Not applicable. This attribute is pending of measurement in additional studies. It is identified as a limitation in the manuscript. |
| Resp9 | Results | Not applicable. This attribute is pending of measurement in additional studies. It is identified as a limitation in the manuscript. |

**Cuestionario de Satisfacción con el tratamiento de la Migraña**

**(Cuestionario Misat-Q)**

Deseamos recoger su opinión sobre **la satisfacción con la medicación** que está tomando **actualmente** para la migraña. Es posible que Ud. esté tomando medicación para tratar más de una enfermedad. Si fuera así, por favor, responda al cuestionario refiriéndose siempre a la medicación que toma **actualmente** para tratar **la migraña**.

En cada pregunta, **tache** el número que mejor refleja su opinión. No existen respuestas correctas o incorrectas. Si no está seguro de alguna de las respuestas, indique la que le parezca más adecuada.

- Ha experimentado algún **efecto adverso** producido por la medicación:

🄋 No, Ninguno ➀ Sí, alguno

| - Esta sección hace referencia a los **efectos no deseados** producidos por el tratamiento que está tomando para la migraña | | | | | | | |
| --- | --- | --- | --- | --- | --- | --- | --- |
|  | No,  Nada | Algo | Ni mucho  ni poco | Bastante | Sí,  Mucho | No procede |  |
| 1. Me molestan los efectos adversos producidos por el tratamiento (control del peso, estado de ánimo, sueño, relaciones sexuales, estreñimiento, fatiga, opresión en el pecho, etc.). | 🄋 | ➀ | ➁ | ➂ | ➃ | ➈ |  |
| 1. Los efectos adversos de la medicación interfieren en mis tareas cotidianas (ir a trabajar, conducir, cuidado familiar, etc.). | 🄋 | ➀ | ➁ | ➂ | ➃ | ➈ |  |
| 1. Los efectos adversos de la medicación interfieren con mi concentración para hacer cosas. | 🄋 | ➀ | ➁ | ➂ | ➃ | ➈ |  |

| - Esta sección se refiere a la **eficacia de la medicación**, es decir, de su capacidad para tratar su migraña y aliviar sus síntomas. | | | | | | |
| --- | --- | --- | --- | --- | --- | --- |
| Durante las **Crisis agudas** | No,  Nada | Algo | Ni mucho  ni poco | Bastante | Sí,  Mucho | No procede |
| 1. La medicación para las crisis que estoy tomando alivia mis síntomas de migraña (dolor de cabeza, náuseas, molestia a la luz y los ruidos, etc.). | 🄋 | ➀ | ➁ | ➂ | ➃ | ➈ |
| 1. Estoy satisfecho con el tiempo que tarda el medicamento en empezar a hacer efecto. | 🄋 | ➀ | ➁ | ➂ | ➃ | ➈ |
| 1. Pienso que, actualmente, controlo bien mis crisis de migraña (su duración, intensidad, visitas a urgencias, etc.). | 🄋 | ➀ | ➁ | ➂ | ➃ | ➈ |

| En la **Prevención** de las crisis | No,  Nada | Algo | Ni mucho  ni poco | Bastante | Sí,  Mucho | No procede |
| --- | --- | --- | --- | --- | --- | --- |
| 1. Gracias al tratamiento que estoy tomando he tenido menos crisis de migraña. | 🄋 | ➀ | ➁ | ➂ | ➃ | ➈ |
| 1. Gracias al tratamiento que estoy tomando las crisis de migraña son menos intensas. | 🄋 | ➀ | ➁ | ➂ | ➃ | ➈ |
| 1. Me encuentro mejor ahora de lo que me encontraba antes de iniciar el tratamiento. | 🄋 | ➀ | ➁ | ➂ | ➃ | ➈ |
| 1. La eficacia de mi tratamiento para prevenir las crisis de migraña me anima a seguir tomándolo. | 🄋 | ➀ | ➁ | ➂ | ➃ | ➈ |

| - Esta sección hace referencia a la **comodidad de la medicación** para la migraña y la facilidad para tomarla. | | | | | |
| --- | --- | --- | --- | --- | --- |
|  | No,  Nada | Algo | Ni mucho  ni poco | Bastante | Sí,  Mucho |
| 1. Me resulta cómoda la forma de administración (oral, liofilizado, inyectada, nasal, intravenosa) de mi medicación para la migraña. | 🄋 | ➀ | ➁ | ➂ | ➃ |
| 1. Es muy raro que olvide tomarme la medicación para la migraña. | 🄋 | ➀ | ➁ | ➂ | ➃ |
| 1. La pauta (horario y número de dosis) de la medicación para la migraña me resultan cómodos. | 🄋 | ➀ | ➁ | ➂ | ➃ |

| - Esta sección hace referencia al **impacto de la medicación** de la migraña en su **vida cotidiana.** | | | | | |
| --- | --- | --- | --- | --- | --- |
|  | No,  Nada | Algo | Ni mucho  ni poco | Bastante | Sí,  Mucho |
| 1. Gracias a la medicación que estoy tomando puedo realizar mejor mis actividades de ocio y tiempo libre. | 🄋 | ➀ | ➁ | ➂ | ➃ |
| 1. Gracias a mi medicación puedo relacionarme mejor con mi familia y amigos. | 🄋 | ➀ | ➁ | ➂ | ➃ |
| 1. Gracias a mi medicación puedo realizar mejor mi trabajo habitual. | 🄋 | ➀ | ➁ | ➂ | ➃ |

| - Esta sección se refiere al **seguimiento de la atención médica** de su migraña. | | | | | |
| --- | --- | --- | --- | --- | --- |
|  | No,  Nada | Algo | Ni mucho  ni poco | Bastante | Sí,  Mucho |
| 1. Mi equipo médico me ha informado adecuadamente acerca de mi migraña y su tratamiento. | 🄋 | ➀ | ➁ | ➂ | ➃ |
| 1. Mi equipo médico me ha informado acerca de los posibles efectos secundarios del tratamiento para mi migraña. | 🄋 | ➀ | ➁ | ➂ | ➃ |
| 1. Mi equipo médico dedica el tiempo necesario para explicar el tratamiento de mi migraña. | 🄋 | ➀ | ➁ | ➂ | ➃ |

| - Para finalizar, se incluyen unas preguntas acerca de su **opinión general** respecto a la medicación para la migraña y su estado de salud. | | | | | |
| --- | --- | --- | --- | --- | --- |
|  | No,  Nada | Algo | Ni mucho  ni poco | Bastante | Sí,  Mucho |
| 1. Estoy convencido de que el tratamiento que estoy tomando para mi migraña es la mejor opción disponible. | 🄋 | ➀ | ➁ | ➂ | ➃ |
| 1. Le aconsejaría a una persona con una migraña como la mía que acudiera al médico para probar el mismo tratamiento. | 🄋 | ➀ | ➁ | ➂ | ➃ |
| 1. En general, me siento satisfecho con el tratamiento para la migraña. | 🄋 | ➀ | ➁ | ➂ | ➃ |

**Gracias por su colaboración**
